# Molecular and cellular mechanisms in diabetic heart failure: Potential therapeutic targets

**DOI:** 10.3389/fendo.2022.947294

**Published:** 2022-09-02

**Authors:** Misganaw Asmamaw Mengstie, Endeshaw Chekol Abebe, Awgichew Behaile Teklemariam, Anemut Tilahun Mulu, Assefa Agegnehu Teshome, Edgeit Abebe Zewde, Zelalem Tilahun Muche, Muluken Teshome Azezew

**Affiliations:** ^1^ Department of Biochemistry, College of Medicine and Health Sciences, Debre Tabor University, Debre Tabor, Ethiopia; ^2^ Department of Anatomy, College of Medicine and Health Sciences, Debre Tabor University, Debre Tabor, Ethiopia; ^3^ Department of Physiology, College of Medicine and Health Sciences, Debre Tabor University, Debre Tabor, Ethiopia

**Keywords:** Diabetes Mellitus, heart failure, Diabetic Heart Failure, mechanisms, therapeutic targets

## Abstract

Diabetes Mellitus (DM) is a worldwide health issue that can lead to a variety of complications. DM is a serious metabolic disorder that causes long-term microvascular and macro-vascular complications, as well as the failure of various organ systems. Diabetes-related cardiovascular diseases (CVD) including heart failure cause significant morbidity and mortality worldwide. Concurrent hypertensive heart disease and/or coronary artery disease have been thought to be the causes of diabetic heart failure in DM patients. However, heart failure is extremely common in DM patients even in the absence of other risk factors such as coronary artery disease and hypertension. The occurrence of diabetes-induced heart failure has recently received a lot of attention. Understanding how diabetes increases the risk of heart failure and how it mediates major cellular and molecular alteration will aid in the development of therapeutics to prevent these changes. Hence, this review aimed to summarize the current knowledge and most recent findings in cellular and molecular mechanisms of diabetes-induced heart failure.

## Introduction

Diabetes Mellitus (DM) is a worldwide health problem characterized by high blood glucose levels caused by a defect in insulin production or insulin resistance, or both (1). DM is a serious metabolic disorder that causes long-term microvascular and macro-vascular complications, as well as the failure of various organ systems (2). Heart Failure (HF) is a long-term condition in which the heart muscles are unable to pump blood effectively enough to meet the body’s needs. It is characterized by cardiomyopathy (cardiac muscle weakness), fibrosis, hypertrophy, cell death, and diastolic dysfunction, followed by systolic dysfunction (3). Uncontrolled DM hurts several systems, including the cardiovascular system. Diabetes-related cardiovascular diseases (CVD) cause significant morbidity and mortality worldwide. Concurrent hypertensive heart disease and/or ischemic coronary artery disease were previously thought to be the cause of HF in diabetic patients. However, even in the absence of predisposing factors for HF, diabetic patients show signs of impaired function and abnormal structure in the heart (4, 5). The occurrence of CVD, including HF in patients with DM, has recently received a lot of attention. According to a plethora of epidemiological evidence, HF is extremely common in DM patients even in the absence of other HF risk factors such as coronary artery disease and hypertension (6–9). HF is four times more prevalent in DM patients than in the general population and is one of the main causes of mortality and morbidity (10). Chronic metabolic imbalance in diabetes can lead to cardiac dysfunction and even HF. Hyperglycemia, insulin resistance, and other factors have been linked to diabetic HF Despite significant advances in our understanding of the pathophysiology of DM-related HF in recent decades, the details of the cellular mechanisms are still not well understood. Understanding how diabetes increases the risk of HF and how it mediates major cellular and molecular alterations will aid in the development of therapeutics to prevent these changes. Hence, this review aimed to summarize the current knowledge and most recent findings in cellular and molecular mechanisms of diabetes-induced HF.

## Molecular and cellular mechanisms of HF in DM

### A brief overview of cardiac metabolism

The regulation of cardiac function, both contractility, and relaxation are dependent on energy metabolism. To maintain its contractile function, the heart needs a continuous and high amount of energy in the form of adenosine 5’-triphosphate (ATP) (11). Under normal conditions, 40-60% of cardiac ATP is produced by the oxidation of fatty acids (FA) in the mitochondria. Glucose is the second most important fuel source for the heart, accounting for 20-40% of cardiac ATP through oxidation (12). Free fatty acids, which originate from either serum albumin or lipoprotein triacylglycerol (TAG) enter the cardiac myocyte through sarcolemma membrane fatty acid transporter proteins, cluster of differentiation-36 (CD36) (13). Once in the cytoplasm, fatty acids can then be used to synthesize a variety of lipid intermediates or can be taken up into the mitochondrial matrix to generate ATP *via* β-oxidation (14). The ability of the heart to switch between available substrates is maintained by tightly regulating fatty acid oxidation at multiple points. The metabolic flexibility of the heart is complex and, it is influenced by several factors such as alteration in contractile work, hormonal change, oxygen supply limitations, and the presence of competing substances (i.e. glucose) (15, 16). Even though fatty acids are the primary fuel source for cardiac cells, they have the highest oxygen requirement to produce ATP and are the least efficient (ATP produced/O2 consumed) myocardial energy substrates. Mitochondrial fatty acid oxidation may also be reduced under stress conditions, resulting in increased glucose utilization (17).

**Figure 1:**
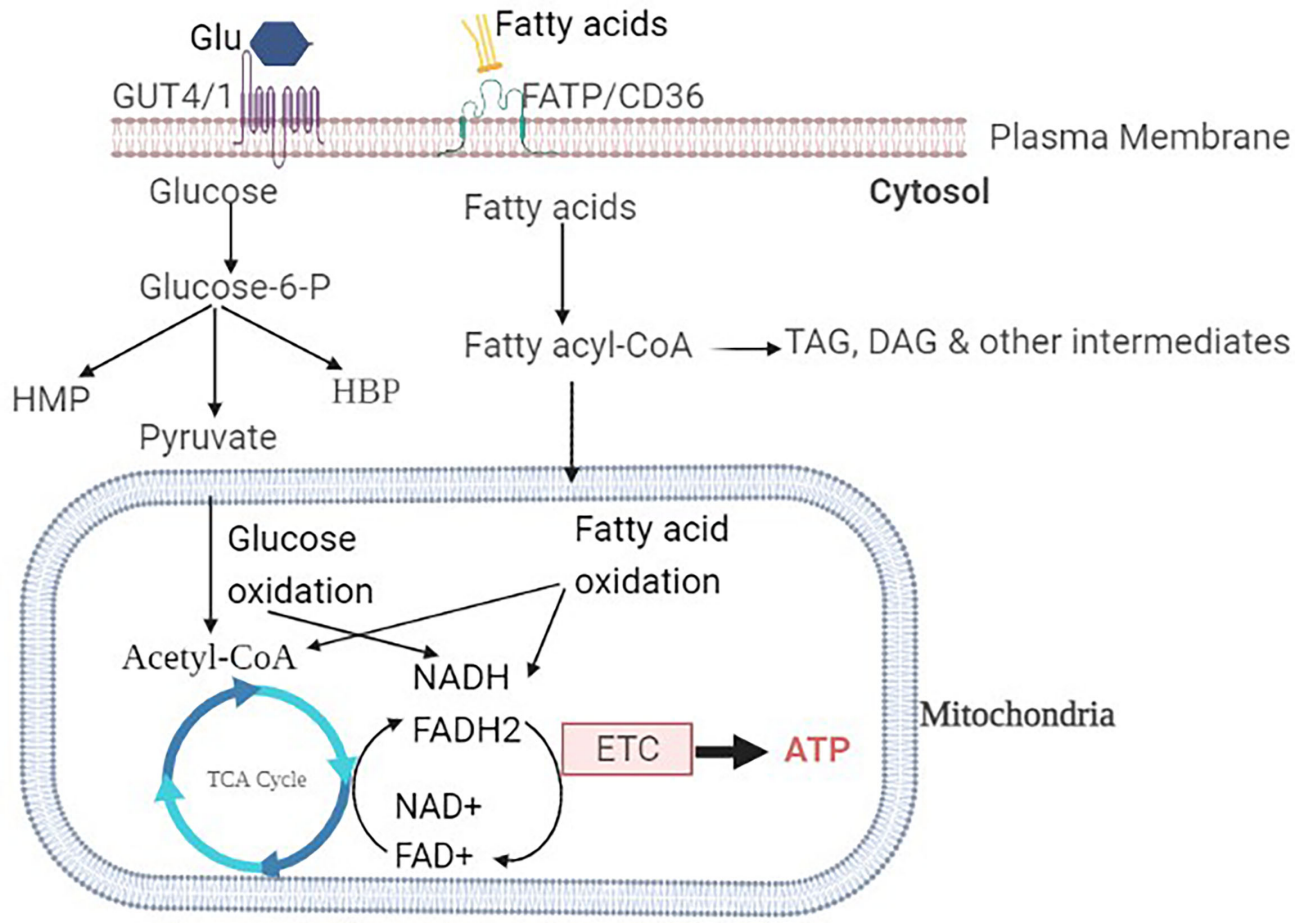
Overview of glucose and fatty acids metabolism in a healthy heart: The glucose transporters GLUT1 and GLUT4 are responsible for glucose uptake into the cardiomyocyte. Once inside the cell, glucose is then phosphorylated to Glucose-6-P. Glucose-6-P is the common intermediate for HMP, HBP, or pyruvate through glycolysis. Pyruvate is then interred to the mitochondria, where it is decarboxylated to acetyl-CoA. Fatty acid transporters (FAT/CD36) transfer non-esterified fatty acids into the cell. Fatty acyl-CoA is formed by intracellular fatty acid activator enzymes and can either be esterified into TAG or reach the mitochondria. Fatty acyl-CoA from mitochondria enters the β-oxidation pathway, where it is converted to acetyl-CoA. Glucose or fatty acid-derived acetyl-CoA enters the TCA cycle, where it undergoes ETC, oxidative phosphorylation, and ATP formation.

The uptake of glucose into the sarcolemma is mediated by insulin-independent glucose transporter type-1 (GLUT-1), insulin-dependent GLUT-4 transporters as the most abundant isoforms. GLUT-4 is the most common isoform in the adult human heart, accounting for 70% of all glucose transporters, whereas GLUT-1 is highly expressed in the fetal heart (18). Insulin is required for the translocation of GLUT-4 from the intracellular compartment to the sarcolemma membrane. Insulin regulates the expression of the GLUT gene, which affects glucose transport, in addition to GLUT translocation (19). Inside cardiac myocytes, glucose can be converted to glucose-6 phosphate by hexokinase or to sorbitol by polyol pathway. As shown in **Figure 1**, glucose-6 phosphate can then be utilized in a variety of metabolic pathways including glycolysis, hexose monophosphate pathway (HMP), and hexosamine biosynthetic pathway (HBP) (20).

### Impaired energy metabolism in diabetic heart

Both glucose and fatty acid metabolic abnormalities have been described leading to a better understanding of the complicated process of HF in DM (4). One of the carbohydrate metabolism abnormalities in DM is the reduced expression of glucose transporters. In a diabetic heart, the expression and translocation of these transporters (GLUT-4) are downregulated (21). For example, atrial GLUT-4 trafficking and expression were impaired in an animal model (streptozotocin-induced type-1 diabetic rodents) (22). Concomitantly, there is the reduction of cardiac glucose uptake, glycolysis, and oxidation associated with a shift towards increased concentration of free fatty acids (accelerated cardiac fatty acid oxidation). A marked increase in cardiac fatty acid oxidation rates and the dominance of fatty acids as the major energy source in the heart is the other significant metabolic change seen in DM (23). Elevated levels of circulating FFAs and their increased oxidation are also inhibitors of both glycolysis and glucose oxidation in the heart. Excess fatty acid oxidation increases mitochondrial oxygen consumption (reduces the pool of oxygen). As a result, the cardiac energy exchange efficiency and energetic reserves are reduced because FFA β-oxidation is less efficient in the limited oxygen availability. During times of increased metabolic demands, this may increase the risk of cardiac dysfunction, including HF (24). Furthermore, the fatty acids taken up by cardiac cells are used for the synthesis of triglycerides (TG) and other lipid intermediates in addition to providing energy. Therefore, cardiac cells’ reliance on fatty acids as an energy source results in a dramatic accumulation of lipids within the myocardium leading to myocardial steatosis, which may contribute to the development of HF (25). These changes have been seen in type-1 and type-2 DM patients, as well as in preclinical models (26–28). The combined myocardial alteration of both glycolysis and fatty acid metabolism has been also observed in many forms of heart disease including HF (29). Moreover, free fatty acids and accumulation of intracellular TG and other lipid intermediates can impair insulin signaling and contribute to cardiac insulin resistance (30). Cardiac insulin resistance, the hallmark of type-2 DM is an independent risk factor for the development of HF (31). Cardiac insulin resistance is linked to decreased cardiac insulin metabolic signaling and is caused by a variety of factors including oxidative stress, hyperglycemia, hyperlipidemia, and dysregulated adipokine/cytokine secretion (32).

### Mitochondrial dysfunction and diabetic HF

**Figure 2:**
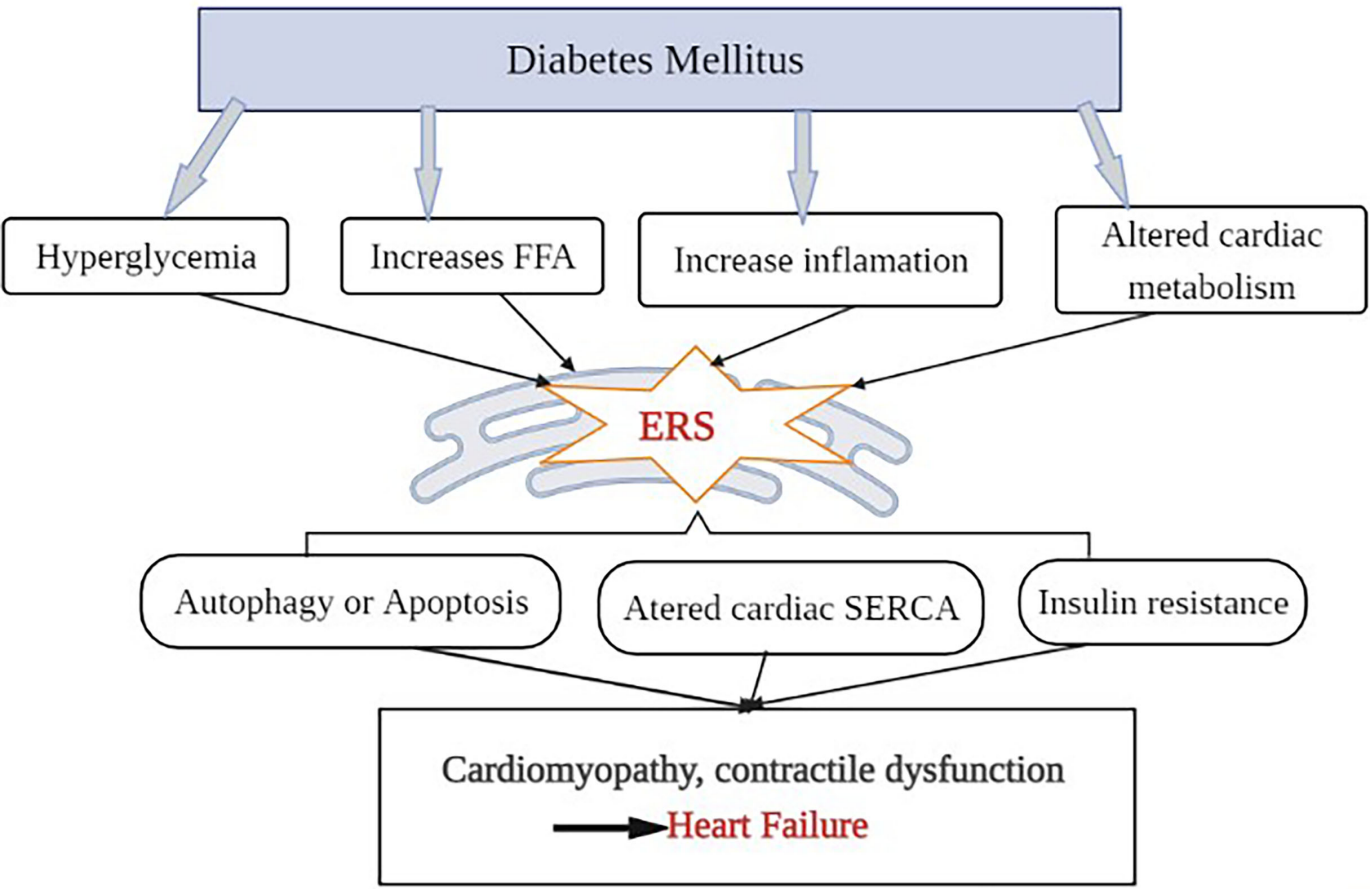
Role of mitochondrial dysfunction in diabetic HF. To maintain ATP production, heart cells in DM adapt to enhanced fatty acid oxidation. Concomitantly, the formation of reactive oxygen species (ROS) is also increased, affecting oxidative phosphorylation, damaging mitochondrial DNA, and disturbing Ca2+ homeostasis. Both mitochondrial DNA damage and mPTP opening (due to Ca^2+^ overload) promote the cardiac apoptotic (programmed cell death) pathway. Through the activation of the cGAS-STING (cyclic GMP-AMP synthase (cGAS) and the cGAS stimulator of interferon genes) pathway, mitochondrial DNA damage may potentially trigger the activation of a pro-inflammatory type of programmed cell death (pyroptosis). Increased mPTP also starts the necroptosis processes. All of which lead to mitochondrial malfunction and ultimately HF.

Mitochondria, the semi-autonomous organelles, and cellular powerhouse play a critical role in ensuring that cells function properly. They are involved in the production of ATP, calcium homeostasis, oxidative stress response, and apoptosis (33). Mitochondrial dysfunction is one of the most principal factors in the development of HF in people with diabetes (34). Cardio myocytes produce about 90% of their ATP through oxidative phosphorylation in the mitochondria (35). As mentioned earlier, cardiac mitochondria in diabetic patients will switch the source of ATP production from glucose to fatty acid oxidation to maintain a constant supply of energy to the cells (36). This process disrupts the oxidative phosphorylation process and causes to produces more reactive oxygen species (ROS) in the electron transport chain. ROS can cause oxidative damage to cellular proteins and lipids. Due to its proximity to the inner membrane, insufficient repair mechanisms, and lack of protective histones, mitochondrial DNA is also extremely vulnerable to ROS (37). ROS also negatively affects myocardial calcium (Ca^2+^) handling resulting in cytosolic Ca^2+^ overload (38). Ca^2+^overload in the cytosol may then cause the opening of the mitochondrial permeability transition pore (mPTP) (39). As illustrated in **Figure 2**, the opening of mPTP, combined with damage to mitochondrial DNA, causes apoptosis (cell death), which leads to cardiac mitochondrial dysfunction and, eventually, HF (40). Recent evidence showed that increased mPTP also initiates the processes of necroptosis (non-programmed cell death) in the diabetic heart (41). Mitochondrial DNA damage could also trigger the activation of a pro-inflammatory type of programmed cell death (pyroptosis) through the activation of the cGAS-STING pathway (42). The cGAS-STING signaling pathway, consisting of the cyclic GMP-AMP synthase (cGAS) and the cyclic GMP-AMP receptor stimulator of interferon genes (STING), is an innate defense mechanism that detects pathogenic DNA. In addition to sensing microbial DNA, cGAS has been proven to detect endogenous DNA, such as DNA released from mitochondria (43). Activation of cardiac pyroptosis promotes pro-inflammatory responses in cardiomyocytes, thereby exacerbating myocardial hypertrophy during the progression of diabetic cardiomyopathy (44). Generally, these forms of cell death could be attributed to the pathogenesis of diabetic HF involving mitochondrial dysfunction. However, various other forms of cell death including, autophagic cell death, autosis, and ferroptosis have been identified and characterized in diabetic cardiomyopathy (45).

### Cardiac endoplasmic reticulum stress (ERS) and diabetic HF

The ER is a vital cell organelle that regulates several homeostatic responses, including lipid and steroid hormone synthesis, calcium homeostasis, secretory and transmembrane protein post-translational modifications, and the folding and maturation of newly synthesized proteins (46). ERS is a chronic perturbation of ER homeostasis marked by the accumulation of unfolded/misfolded proteins caused by a variety of factors including altered glucosylation, nutrient deprivation, oxidative stress, and so on. ERS activates a complex signaling pathway referred to as unfolded protein response (UPR) pathway (47). In normal conditions, UPR activates transcriptional and translational pathways in the ER to reduce the rate of general translation and increase the expression of ER-resident protein chaperones, which helps to restore ER homeostasis (48). Three sensor proteins (normally kept inactive by interacting with ER-resident chaperone proteins) initiate the UPR pathway. These sensor proteins are the kinases PERK (protein kinase R-like ER kinase) and IRE1 (inositol requiring enzyme-1), and the transcription factor ATF-6 (activating transcription factor-6) (49). When these sensors are activated, they cause the transcription of UPR target genes, which leads to a decrease in protein synthesis, an increase in ER chaperone expression, and an increase in proteins involved in ER-associated protein degradation (50). Hence, UPR is the mechanism intended to restore cellular function as a compensatory pro-survival response during ERS. However, a prolonged ER stress response can cause cardiac dysfunction by activating the ER-mediated apoptotic pathway and downstream signaling in the cardio-myocytes (51). Chronic ER stress is associated with metabolic disorders like obesity, diabetes, and age-related pathogenesis, according to growing evidence (52).

**Figure 3:**
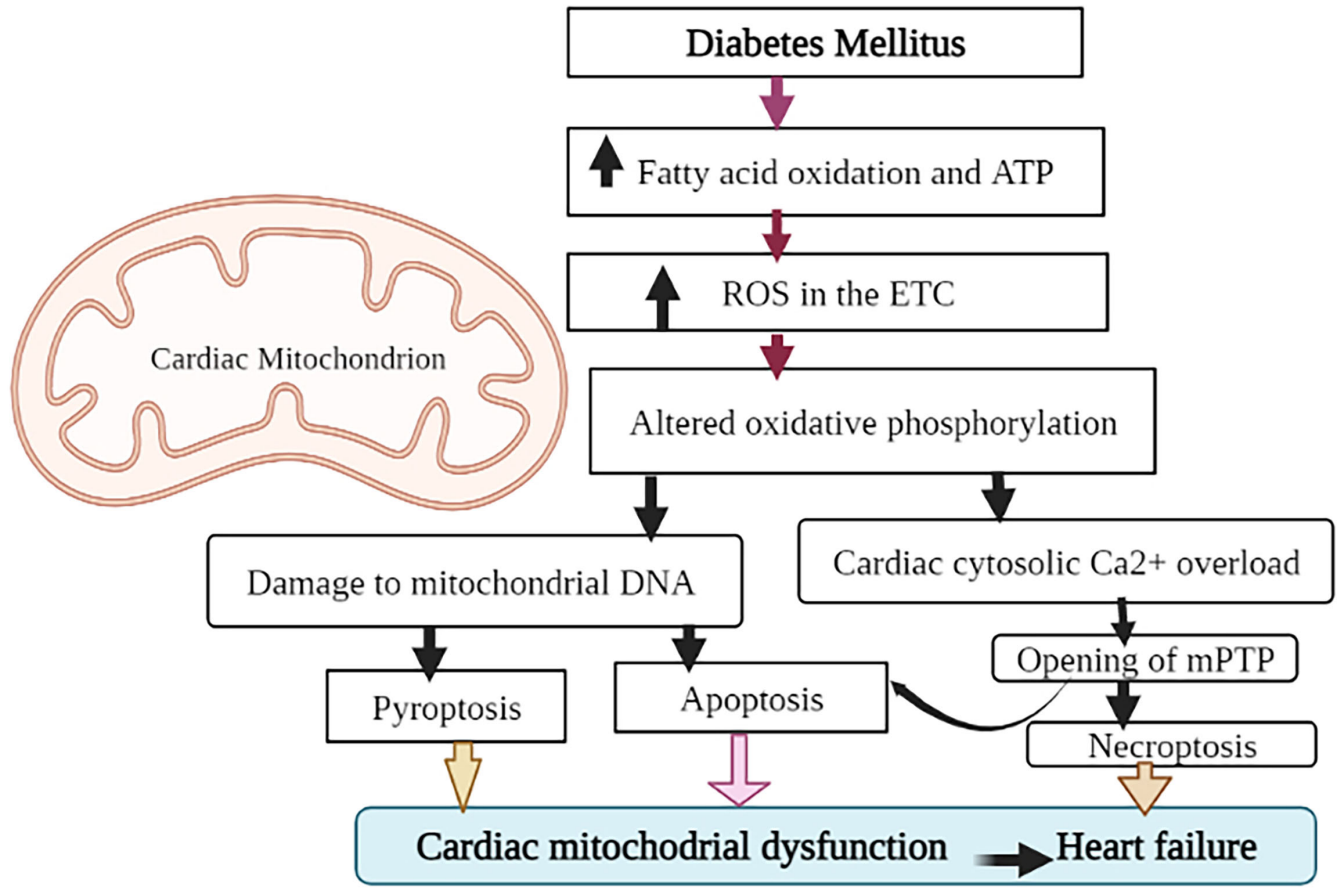
Outlining the role of ER stress in the development of diabetic HF: Diabetes mellitus raises plasma glucose levels, increases the amount of circulating FFAs, alters cardiac metabolism, and triggers an inflammatory response that activates ERS. When ER function is disrupted, it causes cell autophagy or apoptosis, changes in SERCA protein structure and function, and insulin resistance, which leads to diabetic cardiomyopathy, contractile dysfunction, and eventually heart failure.

Cardiac hypertrophy, local inflammation, abnormal intracellular Ca^2+^ handling, oxidative stress, and endothelial dysfunction are all common symptoms of DM (53). DM raises plasma glucose levels, increases FFA levels, activates inflammation, and alters cardiac metabolism and lipotoxicity, all of which contribute to ERS (54). Cardiac ERS then activates UPR and results in myocardial cell apoptosis. In the DM rat model, upregulation of ERS markers and apoptotic molecules have also confirmed myocardial cell apoptosis (55). The other possible mechanism for the ERS-induced HF in diabetes is the alteration of cardiac sarcoplasmic reticulum Ca ^2+^ ATPase (SERCA) protein. The alteration of SERCA in diabetes might be due to hyperglycemia-induced non-enzymatic glycation. SERCA is a membrane-bound intracellular enzyme that transports Ca^2+^ against a concentration gradient by utilizing the free energy of ATP (56). Both human diabetes and experimental animal model studies demonstrated that ERS alters SERCA protein, leading to left ventricular (LV) diastolic dysfunction (57, 58). Reduced cardiac SERCA activity is associated with altered Ca ^2+^ handling and deficient contractility, which can lead to HF (59, 60). Furthermore, studies have also shown that cardiac ERS alters all aspects of cardiac energy metabolism, from substrate utilization to oxidative phosphorylation and energy transport to myofibrils, potentially contributing to HF (61). Overall changes result in contractile dysfunction, left ventricular (LV) hypertrophy, and cardiomyopathy, which together affect cardiac output and eventually lead to HF (62), as shown in **Figure 3**. In general, although more study is needed to fully comprehend the mechanisms underlying these processes, appropriate intervention in the ERS process could be a therapeutic strategy for diabetes-related cardiac complications including HF.

### Role of inflammation in diabetic HF

Systemic chronic inflammation is one of the pathophysiological processes in patients with DM, and it has been a factor in the development of vascular complications (63). Chronic systemic inflammation, caused by hyperglycemia, hyperlipidemia, hyperinsulinemia, and insulin resistance, partially contributes to the development of diabetic HF (64). The accumulation of advanced glycation end products (AGEs), oxidative stress, and hyperlipidemia are followed by activation of the nuclear factor kappa-B (NF-kB) pathway; the renin-angiotensin-aldosterone system (RAAS), and overexpression of inflammatory interleukins are the important molecular mechanisms in the development of diabetic HF (65, 66). Persistent hyperglycemia induces oxidative stress and the accumulation of AGEs, which in turn activates a common signaling pathway involving a transcription factor, known as NF-κB (67). Prolonged hyperglycemia, as previously stated, inhibits glucose oxidation while enhancing fatty acid metabolism in the diabetic heart. Enhanced fatty acid oxidation in the diabetic heart leads to an increase in lipoprotein levels (particularly oxidized LDL is high) (68). It has also been proven that oxidized LDL activates the NF-B signaling pathway (69). NF-κB is a universal transcription factor normally found in an inactive form in the cytoplasm. Once it is activated it trans-locates to the nucleus and is bound with a specific section of DNA that triggers the expression of various cytokines, chemokines, cell adhesion molecules, interleukins, TGFβ, pro-infammatory proteins, and pro-apoptotic genes (70). Finally, the release of those molecules *via* this mechanism causes ROS stress to the myocardium, fibrosis, hypertrophy, cell death, and eventual myocardial diastolic dysfunction, all of which are the early hallmarks of diabetic HF (71). Hence, NF-κB signaling is one of the promising therapeutic targets for diabetic-related chronic complications including diabetic HF (67).

Diabetes can also promote the RAAS system in the heart through several mechanisms. The first is one is hyperglycemia directly stimulates local Angiotensin-II (Ang-II) production in the cardio-myocytes (72). It was proposed that hyperglycemia-induced intracellular generation of Ang-II in the cardiac cells is by chymase proteins. Chymases are a family of serine proteases found primarily in mast cells, fibroblasts, and vascular endothelial cells (73). It was also demonstrated that intracellular Ang-II is correlated with cardiomyocyte apoptosis, oxidative stress, and cardiac fibrosis in diabetic rats (74). Interestingly inhibition of this enzyme has a significant therapeutic advantage in halting the progression of diabetic-induced cardiac and vascular diseases (75, 76). The second mechanism of RAAS activation in the diabetic heart is that high glucose concentrations can enhance the tissue response to Ang-II. This implies that with hyperglycemia, the tissue response to Ang-II can become more contractile. It has also been demonstrated that high glucose concentration augments Ang-II mediated aortic contraction *via* an angiotensin-1 receptor (AT1R) in a rat model (77). The third way by which DM promotes RAAS is through metabolic abnormalities associated with hyperglycemia such as AGEs. AGEs are formed after sustained hyperglycemia, oxidative stress, and dyslipidemia in DM. AGEs could mediate intracellular signaling, gene expression, the release of pro-inflammatory molecules and free radicals, and activation of RAAS through receptor-mediated signaling cascade (78, 79). Furthermore, AGEs activate the chymase-dependent production of Ang-II (80). Ang-II plays a significant role in regulating various physiological processes. It acts as a potent vasoconstrictor, promotes growth factors, migration, proliferation, and hypertrophy of vascular smooth muscle cells and cardiac fibroblasts, and increases catecholamine release. Although these mechanisms initially serve to maintain cardiac output, they eventually contribute to the progression of HF (81).

### Role of autophagy in diabetic HF

Autophagy is the general term that refers to the process involving the decomposition of intracellular components *via* lysosomes. It is crucial to all types of cells including cardiovascular cells for maintaining homeostasis and preventing nutritional, metabolic, and infection-related stress (82). Micro-autophagy, macro-autophagy, and chaperone-mediated autophagy (CMA) are the three main types of autophagy in mammalian cells, based on the mode of cargo delivery to the lysosome (83). To capture the cargo in micro-autophagy, direct invaginations of the lysosomal membrane or vacuolar membranes are used. CMA is a type of autophagy that uses chaperones to selectively degrade soluble cytosolic proteins in the lysosomes. Macro-autophagy (hereafter referred to as autophagy) is the most extensively studied type of autophagy characterized by the sequestration of cytoplasmic materials in double-membrane vacuoles called auto-phagosomes, which are then delivered to the lysosome for degradation (84, 85). Several principal factors control this intracellular degradation process. Autophagy-related (ATG) genes and proteins play a crucial role in the process of autophagy such as induction, cargo selection, auto-phagosome formation, fusion, and degradation. To date, more than 30 ATG genes and proteins have been discovered (86). Although there are several regulatory pathways for cellular autophagy, the mammalian target of rapamycin (mTOR) pathway and adenosine 5’monophosphate-activated protein kinase (AMPK) are the most important inhibitors and activators, respectively (87). mTOR is a serine/threonine kinase that is highly conserved from yeast to humans (88). By phosphorylating complex components such as ATG13 and ATG1 (the mammalian ULK1/2 homolog), mTOR inhibits the autophagy-initiating UNC-5 like autophagy activating kinase (ULK) complex. This disrupts the interaction between ATG1 and ATG13, preventing the interaction and phosphorylation of ULK1 by AMPK, and finally inhibiting autophagosome initiation (88, 89). On the other hand, AMPK (a nutrient sensor) is the major activator of autophagy. AMPK promotes autophagy either directly by phosphorylating autophagy-related proteins in the mTOR, such as ULK, or indirectly by regulating autophagy-related gene expression downstream of transcription factors (90).

Although autophagy is important for maintaining cellular homeostasis in healthy cardiac tissues by preventing the accumulation of dysfunctional organelles and cytotoxic protein aggregates, some recent research has found that this process is frequently impaired in diabetic hearts (91). There are contradictory evidences regarding whether autophagy flux is upregulated or downregulated in the hearts of DM patients. Some studies have tried to distinguish autophagy adaptation in the heart of type 1 and type 2 DM. For instance, in animal models, autophagy flux was found to be enhanced in the heart of type 1 diabetics but suppressed in type 2 diabetics (92). Because GLUT-4 and FAT/CD36 cannot translocate to the membrane due to insulin deficiency in type 1 DM, glucose and fatty acid uptake by the membrane is prevented, putting the cell into a state of nutrient deprivation. AMPK is activated by ATP depletion or AMP accumulation. As a result, in type 1 diabetic hearts, autophagy flux is enhanced (93, 94). Diastolic dysfunction and pathogenesis in diabetic cardiomyopathy have been linked to autophagy upregulation in the hearts of type 1 diabetic (95). In contrast, some experimental studies revealed that diminished autophagy in type 1 DM is an adaptive response that limits cardiac dysfunction (96, 97). Unlike type 1 DM, autophagy would be expected to be suppressed in type 2 diabetic hearts (98). Although the exact molecular mechanism is unknown, AMPK must play a major role in the suppression of autophagy in type 2 DM. Overeating and/or obesity, common features of type 2 DM are possible causes that downregulate AMPK activity by increasing intracellular levels of energy substrates like glucose and/or FFA, thereby suppressing autophagy (99). Suppressing AMPK signaling lowers cardiac glucose transport, glycolysis, and fatty acid oxidation, as well as the energy supply required for cardiac function. Indeed, downregulation of AMPK activity is a key factor in the development of diabetic HF in type 2 DM (92). It was also demonstrated that a reduction in cardiac autophagy and the subsequent decrease of cell viability was implicated in cardiac dysfunction in the diabetic heart (78). However, still, there are some contradictory pieces of evidence, which demonstrated that suppression of autophagy in diabetes is protective against cardiac cell dysfunction and HF too (100, 101). Therefore, further study is needed to determine the exact role of autophagy in type 1 or type 2 diabetic HF, as well as whether the response of autophagy in the diabetic heart is protective or not.

### Role of epigenetics in diabetic HF

Epigenetics is the study of heritable changes in gene expression patterns that are not linked to changes in DNA sequence. DNA methylation and histone modification are the key epigenetic processes that control gene expression (102). Epigenetic dysregulation has a role in the development of many diseases including DM. DNA methylation is a biological event that occurs when DNA methyltransferases add methyl groups to DNA nucleotides (cytosine or adenine). It is usually exerted at the 5′-position of cytosine residues in CpG dinucleotides. DNA methylation regulates gene expression by preventing transcription factors from binding to DNA (103). Recently increasing evidence showed that DNA methylation plays a critical role in the pathogenesis of diabetic complications including HF (104). It was discovered that tumor necrosis factor- α (TNF-α) boosts the SERCA promotor region by enhancing DNA methyltransferase activity in diabetic hyperglycemia. In cardiomyocytes, the expression of the protein is reduced when the SERCA gene is methylated (105). As previously indicated, downregulation of SERCA expression might result in diastolic dysfunction and contractility impairment, which can contribute to diabetic HF (106). Furthermore, a positive correlation was observed between the severity of HF and the expression level of TNF-α (107). This is also supported by the study that aberrant DNA methylation in human cardiomyocytes impairs cardiac contractility, and causes mitochondrial damage, as well as lipid and glucose metabolic problems (108). Taken together, these studies suggest that DNA methylation plays a role in the adverse cardiac remodeling associated with the development of diabetic HF, albeit more research is needed.

Histone modification is another epigenetic process have been also identified as a key contributor to the development of diabetes-related HF (109). Together with DNA, four types of histone proteins (H2A, H2B, H3, and H4) make up the fundamental unit of chromatin known as the nucleosome. Histone modification is an epigenetic process in which histone proteins are methylated, acetylated, phosphorylated, adenylated, ubiquitinated, or ADP ribosylated (110). The transcriptional activity of associated genes is affected by these modifications. Histone modifications also affect the binding ability of other proteins to histones *via* altering local hydrophobicity, RNA polymerase status, and binding affinity for other transcription coactivators (111). In diabetic myocardium, hyperglycemia promotes the production of mitochondrial ROS, which in turn induces JunD (Jund proto-oncogene subunit) downregulation. Since JunD is one of the oxidative stress gatekeepers, its downregulation causes cardiac dysfunction in both experimental and human DM investigations (112). In vascular cells, ROS also causes long-term epigenetic activation of the NF-B subunit p65. The methylation of histone 3 on lysine 4 has been identified as the epigenetic alteration that enhances p65 gene expression and consequent pro-inflammatory gene expression. These findings show that hyperglycemia-induced histone alteration could be a separate risk factor for diabetes complications including HF (113). Recent evidence from *in vitro* and *in vivo* revealed that Histone deacetylation appears to play a role in the etiology of diabetes complications, including HF. So, it has been proposed that the balance between histone acetylation and deacetylation must be tightly regulated (114). Hence, epigenetics in cardiovascular research and therapy is an exciting and promising emerging research field (115).

### Role of microRNAs in diabetic HF

MicroRNAs (miRNAs) are endogenous, noncoding, single-strand RNAs with an average length of 22 nucleotides that regulate gene expression. miRNAs control gene expression in two ways: by inhibiting translation or by promoting the degradation of target messenger RNA (mRNA) (116). Studies have demonstrated that the expression level of miRNA in the heart of DM patients was found to be different when compared with healthy individuals (117). Changes in miRNA synthesis and levels have been linked to cardiac remodeling and the development of diabetic HF (118). The miRNAs may target mitochondrial function, ROS generation, Ca2+ perturbation, apoptosis, and fibrosis are all thought to be significant pathways for cardiac hypertrophy, remodeling, and HF development (119). One of the distinctive structural characteristics of early HF is cardiomyocyte hypertrophy. Numerous miRNAs were shown to be dysregulated and contributed to the pathophysiology of cardiomyocyte hypertrophy in DM (120). For instance, miR-133 is highly expressed in heart tissue and is known to be involved in diabetic cardiac hypertrophy (121). According to a study done on type 2 DM patients, miR-17 expression was up-regulated, while miR-24, miR-150, miR-199a, miR-214, and miR-320a expression was down-regulated in the diabetic cardiomyopathy patients compared to healthy controls. And it was concluded that dysregulation of circulating miRNA may contribute to the pathogenesis of diabetic induced-cardiomyopathy (122). It has also been demonstrated that upregulation of nuclear miR-320 mediates diabetes-induced cardiac dysfunction by activating the transcription of fatty acid metabolic genes, causing lipotoxicity in the hearts of diabetic mice (123). As a result, it has recently been suggested that measuring circulating miRNAs in combination with other biological markers could substantially improve the diagnostic potential of detecting diabetic cardiomyopathy (124). A significant reduction of miR-21 was also observed in the diastolic dysfunctional heart of diabetic mice. And the delivery of miR-21 efficiently protected against cardiac diastolic dysfunction (one of the early manifestations of HF) by targeting gelsolin protein (125). Furthermore, miRNA can influence other epigenetic changes such as histone H2A mRNA levels, histone deacetylase, and DNA methyltransferases, resulting in gene modification and expression modulation (126). However, the precise involvement of miRNAs in the development of diabetic HF, as well as their cross-talk with other epigenetic processes, is not clearly elucidated.

## Conclusions

Metabolic impairment, ERS, autophagy, the inflammatory process, epigenetics, and microRNAs are just a few of the cellular and molecular mechanisms that have been proposed and explored in diabetic HF. As a result, limiting cardiac fatty acid oxidation, boosting glucose oxidation, mitigating ERS, and moderating inflammation could all be promising therapeutic approaches for diabetic HF. However, the role of some key pathways, such as autophagy, epigenetic mechanisms, and microRNAs in the pathogenesis of diabetic HF, are still under-studied and require further investigation.

## Author contributions

MM developed the concept of the review. The manuscript of the protocol was drafted by EA, ATek, AM, ATes, EZ, and ZM then critically revised by MM and MA. MM developed and provided feedback for all sections of the review protocol. All authors have approved the final version of the manuscript to be published.

## Conflict of interest

The authors declare that the research was conducted in the absence of any commercial or financial relationships that could be construed as a potential conflict of interest.

## Publisher’s note

All claims expressed in this article are solely those of the authors and do not necessarily represent those of their affiliated organizations, or those of the publisher, the editors and the reviewers. Any product that may be evaluated in this article, or claim that may be made by its manufacturer, is not guaranteed or endorsed by the publisher.

## Abbreviations

ATP, adenosine triphosphate; CD36, cluster of differentiation; CVD, cardiovascular diseases; DM, diabetes mellitus; ERS, endoplasmic reticulum stress; ETC, electron transport chain; GLUT, glucose transporters; HBP, hexosamine biosynthetic pathway; HF, heart failure; HMP, hexose monophosphate pathway; miRNA, microRNAs; mPTP, mitochondrial permeability transition pore; mRNA, messenger RNA; ROS, reactive oxygen species; SERCA, sarcoplasmic reticulum calcium ATPase; TAG, triacylglycerol; UPR, unfolded protein response.

## References

[1] Al-Lawati JA. Diabetes mellitus: A local and global public health emergency! Oman Med J (2017) 32(3):177–9. doi: 10.5001/omj.2017.34 PMC5447787 28584596

[2] Chawla A Chawla R Jaggi S. Microvasular and macrovascular complications in diabetes mellitus: Distinct or continuum? Indian J Endocrinol Metab (2016) 20(4):546–53. doi: 10.4103/2230-8210.183480 PMC4911847 27366724

[3] Jia G Hill MA Sowers JR. Diabetic cardiomyopathy: An update of mechanisms contributing to this clinical entity. Circ Res (2018) 122(4):624–38. doi: 10.1161/CIRCRESAHA.117.311586 PMC5819359 29449364

[4] Kenny HC Abel ED. Heart failure in type 2 diabetes mellitus: Impact of glucose-lowering agents, heart failure therapies, and novel therapeutic strategies. Circ Res (2019) 124(1):121–41. doi: 10.1161/CIRCRESAHA.118.311371 PMC6447311 30605420

[5] Giugliano D Meier JJ Esposito K. Heart failure and type 2 diabetes: From cardiovascular outcome trials, with hope. Diabetes Obes Metab (2019) 21(5):1081–7. doi: 10.1111/dom.13629 30609236

[6] Dunlay SM Givertz MM Aguilar D Allen LA Chan M Desai AS. Type 2 diabetes mellitus and heart failure a scientific statement from the American heart association and the heart failure society of America. Circulation (2019) 140:294–324. doi: 10.1161/cir.0000000000000691 31167558

[7] Thomas MC. Type 2 diabetes and heart failure: Challenges and solutions. Curr Cardiol Rev (2016) 12:249–55. doi: 10.2174/1573403X12666160606120254 PMC5011193 27280301

[8] Schütt KA. Heart failure in patients with diabetes. Diabetologe (2021) 17(5):607–17. doi: 10.1007/s11428-021-00752-w

[9] Oo MM Tan Chung Zhen I Ng KS Tan KL Tan ATB Vethakkan SR. Observational study investigating the prevalence of asymptomatic stage b heart failure in patients with type 2 diabetes who are not known to have coronary artery disease. BMJ Open (2021) 11(1):1–8. doi: 10.1136/bmjopen-2020-039869 PMC7825254 33478961

[10] Ceriello A Catrinoiu D Chandramouli C Cosentino F Dombrowsky AC Itzhak B. Heart failure in type 2 diabetes: Current perspectives on screening, diagnosis and management. Cardiovasc Diabetol (2021) 20(1):1–19. doi: 10.1186/s12933-021-01408-1 34740359 PMC8571004

[11] Martínez MS García A Luzardo E Chávez-Castillo M Olivar LC Salazar J. Energetic metabolism in cardiomyocytes: Molecular basis of heart ischemia and arrhythmogenesis. Vessel Plus (2017) 1:230–41. doi: 10.20517/2574-1209.2017.34

[12] Hausner EA Elmore SA Yang X. Overview of the components of cardiac metabolism. Drug Metab Dispos (2019) 47(6):673–88. doi: 10.1124/dmd.119.086611 PMC7333657 30967471

[13] Glatz JFC Luiken JJFP Nabben M. Cd36 (Sr-b2) as a target to treat lipid overload-induced cardiac dysfunction. J Lipid Atheroscler (2020) 9(1):66–78. doi: 10.12997/jla.2020.9.1.66 32821722 PMC7379071

[14] Schulze PC Konstantinos Drosatos IJG. Lipid use and misuse by the heart. Circ Res (2017) 118(11):1736–51. doi: 10.1161/circresaha.116.306842 PMC5340419 27230639

[15] Angelini A Pi X Xie L. Dioxygen and metabolism; dangerous liaisons in cardiac function and disease. Front Physiol (2017) 8(1044):1–20. doi: 10.3389/fphys.2017.01044 29311974 PMC5732914

[16] Karwi QG Uddin GM Ho KL Lopaschuk GD. Loss of metabolic flexibility in the failing heart. Front Cardiovasc Med (2018) 5(68):1–19. doi: 10.3389/fcvm.2018.00068 29928647 PMC5997788

[17] Pascual F Coleman RA. Fuel availability and fate in cardiac metabolism: A tale of two substrates. Biochim Biophys Acta (2017) 1860(10):1425–33. doi: 10.1016/j.bbalip.2016.03.014 PMC4983230 26993579

[18] Varma U Koutsifeli P Benson VL Mellor KM Delbridge LMD. Molecular mechanisms of cardiac pathology in diabetes – experimental insights. Biochim Biophys Acta - Mol Basis Dis (2018) 1864(5):1949–59. doi: 10.1016/j.bbadis.2017.10.035 29109032

[19] Wang T Wang J Hu X Xian-Ju H Guo-Xun C. Current understanding of glucose transporter 4 expression and functional mechanisms. World J Biol Chem (2020) 11(3):76–118. doi: 10.4331/wjbc.v11.i3.76 33274014 PMC7672939

[20] Tran DH Wang ZV. Glucose metabolism in cardiac hypertrophy and heart failure. J Am Heart Assoc (2019) 8(12):e012673. doi: 10.1161/JAHA.119.012673 31185774 PMC6645632

[21] Szablewski L. Glucose transporters in healthy heart and in cardiac disease. Int J Cardiol [Internet] (2017) 230:70–5. doi: 10.1016/j.ijcard.2016.12.083 28034463

[22] Maria Z Campolo AR Lacombe VA. Diabetes alters the expression and translocation of the insulin-sensitive glucose transporters 4 and 8 in the atria. PloS One (2015) 10(12):e0146033. doi: 10.1371/journal.pone.0146033 26720696 PMC4697822

[23] Karwi QG Sun Q Lopaschuk GD. The contribution of cardiac fatty acid oxidation to diabetic cardiomyopathy severity. Cells (2021) 10(11):3259. doi: 10.3390/cells10113259 34831481 PMC8621814

[24] Kota SK Kota SK Jammula S Panda S Modi KD. Effect of diabetes on alteration of metabolism in cardiac myocytes: Therapeutic implications. Diabetes Technol Ther (2011) 13(11):1155–60. doi: 10.1089/dia.2011.0120 21751873

[25] Yamamoto T Sano M. Deranged myocardial fatty acid metabolism in heart failure. Int J Mol Sci (2022) 23(2):996. doi: 10.3390/ijms23020996 35055179 PMC8779056

[26] Malfitano C de Souza Junior AL Carbonaro M Bolsoni-Lopes A Figueroa D de Souza LE. Glucose and fatty acid metabolism in infarcted heart from streptozotocin-induced diabetic rats after 2weeks of tissue remodeling. Cardiovasc Diabetol (2015) 14(1):1–10. doi: 10.1186/s12933-015-0308-y 26553117 PMC4640361

[27] Amaral N Okonko DO. Metabolic abnormalities of the heart in type II diabetes. Diabetes Vasc Dis Res (2015) 12(4):239–48. doi: 10.1177/1479164115580936 25941161

[28] Herrero P Peterson LR McGill JB Matthew S Lesniak D Dence C. Increased myocardial fatty acid metabolism in patients with type 1 diabetes mellitus. J Am Coll Cardiol (2006) 47(3):598–604. doi: 10.1016/j.jacc.2005.09.030 16458143

[29] Lopaschuk GD Karwi QG Tian R Wende AR Abel ED. Cardiac energy metabolism in heart failure. Circ Res (2021) 128(10):1487–513. doi: 10.1161/CIRCRESAHA.121.318241 PMC8136750 33983836

[30] Nakamura M Sadoshima J. Cardiomyopathy in obesity, insulin resistance and diabetes. J Physiol (2020) 598(14):2977–93. doi: 10.1113/JP276747 30869158

[31] Zheng L Li B Lin S Chen L Li H. Role and mechanism of cardiac insulin resistance in occurrence of heart failure caused by myocardial hypertrophy. Aging (Albany NY) (2019) 11(16):6584–90. doi: 10.18632/aging.102212 PMC6738426 31461405

[32] Aroor AR Chirag H. Mandavia and JRS. insulin resistance and heart failure. Curr Heart Fail Rep (2012) 8(4):609–17. doi: 10.1016/j.hfc.2012.06.005 PMC3457065 22999243

[33] Li A Gao M Jiang W Qin Y Gong G. Mitochondrial dynamics in adult cardiomyocytes and heart diseases. Front Cell Dev Biol (2020) 8:1555. doi: 10.3389/fcell.2020.584800 PMC7773778 33392184

[34] Jubaidi FF Zainalabidin S Mariappan V Budin SB. Mitochondrial dysfunction in diabetic cardiomyopathy: The possible therapeutic roles of phenolic acids. Int J Mol Sci (2020) 21(17):6043. doi: 10.3390/ijms21176043 PMC7503847 32842567

[35] Dorn GW Vega RB Kelly DP. Mitochondrial biogenesis and dynamics in the developing and diseased heart. Genes Dev (2015) 29(19):31–51. doi: 10.1101/gad.269894.115 PMC4604339 26443844

[36] Lopaschuk GD. Fatty acid oxidation and its relation with insulin resistance and associated disorders. Ann Nutr Metab (2016) 68(3):15–20. doi: 10.1159/000448357 27931032

[37] Gollmer J Zirlik A Bugger H. Mitochondrial mechanisms in diabetic cardiomyopathy. Diabetes Metab J (2020) 44(1):33–53. doi: 10.4093/dmj.2019.0185 32097997 PMC7043970

[38] Münzel T Camici GG Maack C Bonetti NR Fuster V Kovacic JC. Impact of oxidative stress on the heart and vasculature: Part 2 of a 3-part series. J Am Coll Cardiol (2017) 70(2):212–29. doi: 10.1016/j.jacc.2017.05.035 PMC5663297 28683969

[39] Hurst S Hoek J Sheu S-S. Mitochondrial Ca2+ and regulation of the permeability transition pore. J Bioenerg Biomembr (2017) 49:27–47. doi: 10.1007/s10863-016-9672-x 27497945 PMC5393273

[40] Davidson SM Adameová A Barile L Cabrera-Fuentes HA Lazou A Pagliaro P. Mitochondrial and mitochondrial-independent pathways of myocardial cell death during ischaemia and reperfusion injury. J Cell Mol Med (2020) 24(7):3795–806. doi: 10.1111/jcmm.15127 PMC7171390 32155321

[41] Bonora M Patergnani S Ramaccini D Morciano G Pedriali G Kahsay AE. Physiopathology of the permeability transition pore: Molecular mechanisms in human pathology. Biomolecules (2020) 10(7):1–25. doi: 10.3390/biom10070998 PMC7408088 32635556

[42] Yan M Li Y Luo Q Zeng W Shao X Li L. Mitochondrial damage and activation of the cytosolic DNA sensor cGAS–STING pathway lead to cardiac pyroptosis and hypertrophy in diabetic cardiomyopathy mice. Cell Death Discov (2022) 8(1):1–12. doi: 10.1038/s41420-022-01046-w 35538059 PMC9091247

[43] Ou L Zhang A Cheng Y Chen Y. The cGAS-STING pathway: A promising immunotherapy target. Front Immunol (2021) 12:1–15. doi: 10.3389/fimmu.2021.795048 PMC8695770 34956229

[44] Cai Z Yuan S Luan X Feng J Deng L Zuo Y. Pyroptosis-related inflammasome pathway: A new therapeutic target for diabetic cardiomyopathy. Front Pharmacol (2022) 13:1–11. doi: 10.3389/fphar.2022.842313 PMC8959892 35355717

[45] Wei J Zhao Y Liang H Du W Wang L. Preliminary evidence for the presence of multiple forms of cell death in diabetes cardiomyopathy. Acta Pharm Sin B [Internet] (2022) 12(1):1–17. doi: 10.1016/j.apsb.2021.08.026 PMC8799881 35127369

[46] Schwarz DS Blower MD. The endoplasmic reticulum: Structure, function and response to cellular signaling. Cell Mol Life Sci (2016) 73(1):79–94. doi: 10.1007/s00018-015-2052-6 26433683 PMC4700099

[47] Liu MQ Chen Z Chen LX. Endoplasmic reticulum stress: A novel mechanism and therapeutic target for cardiovascular diseases. Acta Pharmacol Sin [Internet] (2016) 37(4):425–43. doi: 10.1038/aps.2015.145 PMC4820795 26838072

[48] Sicari D Delaunay-Moisan A Combettes L Chevet E Igbaria A. A guide to assessing endoplasmic reticulum homeostasis and stress in mammalian systems. FEBS J (2020) 287(1):27–42. doi: 10.1111/febs.15107 31647176

[49] Gardner BM Pincus D Gotthardt K Gallagher CM Walter P. Endoplasmic reticulum stress sensing in the unfolded protein response. Cold Spring Harb Perspect Biol (2013) 5(3):1–15. doi: 10.1101/cshperspect.a013169 PMC3578356 23388626

[50] Adams CJ Kopp MC Larburu N Nowak PR Ali MMU. Structure and molecular mechanism of ER stress signaling by the unfolded protein response signal activator IRE1. Front Mol Biosci (2019) 6(11):1–12. doi: 10.3389/fmolb.2019.00011 30931312 PMC6423427

[51] Wang S Binder P Fang Q Wang Z Xiao W Liu W. Endoplasmic reticulum stress in the heart: Insights into mechanisms and drug targets. Br J Pharmacol (2018) 175(8):1293–304. doi: 10.1111/bph.13888 PMC5867005 28548229

[52] Bhattarai KR Chaudhary M Kim HR Chae HJ. Endoplasmic reticulum (ER) stress response failure in diseases. Trends Cell Biol (2020) 30(9):672–5. doi: 10.1016/j.tcb.2020.05.004 32561138

[53] Chen Y Zhao X Wu H. Metabolic stress and cardiovascular disease in diabetes mellitus: The role of protein O -GlcNAc modification. Arterioscler Thromb Vasc Biol (2019) 39(10):1911–24. doi: 10.1161/ATVBAHA.119.312192 PMC6761006 31462094

[54] Ruan Y Zeng J Jin Q Chu M Ji K Wang Z. Endoplasmic reticulum stress serves an important role in cardiac ischemia/reperfusion injury (Review). Exp Ther Med (2020) 20(6):1–1. doi: 10.3892/etm.2020.9398 33199993 PMC7664614

[55] Yang Q Gao H Dong R Wu YQ. Sequential changes of endoplasmic reticulum stress and apoptosis in myocardial fibrosis of diabetes mellitus-induced rats. Mol Med Rep (2016) 13(6):5037–44. doi: 10.3892/mmr.2016.5180 PMC4878574 27121167

[56] Horáková L Strosova MK Spickett CM Blaskovic D. Impairment of calcium ATPases by high glucose and potential pharmacological protection. Free Radic Res (2013) 47(1):81–92. doi: 10.3109/10715762.2013.807923 23710650

[57] Takada A Miki T Kuno A Kouzu H Sunaga D Itoh T. Role of ER stress in ventricular contractile dysfunction in type 2 diabetes. PloS One (2012) 7(6):e39893. doi: 10.1371/journal.pone.0039893 22768157 PMC3387241

[58] Lee Y Chakraborty S Muthuchamy M. Roles of sarcoplasmic reticulum Ca2+ ATPase pump in the impairments of lymphatic contractile activity in a metabolic syndrome rat model. Sci Rep [Internet] (2020) 10(1):1–20. doi: 10.1038/s41598-020-69196-4 PMC7378550 32704072

[59] Zhihao L Jingyu N Lan L Michael S Rui G Xiyun B. SERCA2a: A key protein in the Ca2+ cycle of the heart failure. Heart Fail Rev (2020) 25(3):523–35. doi: 10.1007/s10741-019-09873-3 31701344

[60] Wang L Myles RC Lee IJ Bers DM Ripplinger CM. Role of reduced sarco-endoplasmic reticulum Ca2+-ATPase function on sarcoplasmic reticulum Ca2+ alternans in the intact rabbit heart. Front Physiol (2021) 12:1–11. doi: 10.3389/fphys.2021.656516 PMC8144333 34045974

[61] Prola A Nichtova Z Pires Da Silva J Piquereau J Monceaux K Guilbert A. Endoplasmic reticulum stress induces cardiac dysfunction through architectural modifications and alteration of mitochondrial function in cardiomyocytes. Cardiovasc Res (2019) 115(2):328–42. doi: 10.1093/cvr/cvy197 30084984

[62] Palomer X Pizarro-Delgado J Vázquez-Carrera M. Emerging actors in diabetic cardiomyopathy: Heartbreaker biomarkers or therapeutic targets? Trends Pharmacol Sci (2018) 39(5):452–67. doi: 10.1016/j.tips.2018.02.010 29605388

[63] Tsalamandris S Antonopoulos AS Oikonomou E Papamikroulis GA Vogiatzi G Papaioannou S. The role of inflammation in diabetes: Current concepts and future perspectives. Eur Cardiol Rev (2019) 14(1):50–9. doi: 10.15420/ecr.2018.33.1 PMC6523054 31131037

[64] Kaur N Guan Y Raja R Ruiz-Velasco A Liu W. Mechanisms and therapeutic prospects of diabetic cardiomyopathy through the inflammatory response. Front Physiol (2021) 12:1–11. doi: 10.3389/fphys.2021.694864 PMC8257042 34234695

[65] Senatus L Maclean M Arivazhagan L Egaña-gorroño L López-díez R Manigrasso MB. Inflammation meets metabolism: Roles for the receptor for advanced glycation end products axis in cardiovascular disease. Immunometabolism (2021) 3(3):1–23. doi: 10.20900/immunometab20210024 PMC8232874 34178389

[66] Ramesh P Yeo JL Brandy EM McCAnn GP. Role of inflammation in diabetic cardiomyopathy. Ther Adv Endocrinol Metab (2022) 13:1–13. doi: 10.1177/20420188221083530 PMC8928358 35308180

[67] Suryavanshi SV Kulkarni YA. NF-κβ: A potential target in the management of vascular complications of diabetes. Front Pharmacol (2017) 8(7):1–12.10.3389/fphar.2017.00798 29163178 10.3389/fphar.2017.00798 PMC5681994

[68] Bonilha I Hajduch E Luchiari B Nadruz W Le GW Sposito AC. The reciprocal relationship between LDL metabolism and type 2 diabetes mellitus. Metabolites (2021) 11(12):807. doi: 10.3390/metabo11120807 34940565 PMC8708656

[69] Quagliariello V Bonelli A Paccone A Buccolo S Iovine M Botti G. Oxidized low-density lipoproteins increases nivolumab-induced cardiotoxicity through TLR4/NF-KB and NLRP3 pathways. Eur Heart J (2021) 42:2837. doi: 10.1093/eurheartj/ehab724.2837

[70] Verboom L Hoste E van Loo G. OTULIN in NF-κB signaling, cell death, and disease. Trends Immunol (2021) 42(7):590–603. doi: 10.1016/j.it.2021.05.003 34074601

[71] Yu H Lin L Zhang Z Zhang H Hu H. Targeting NF-κB pathway for the therapy of diseases: Mechanism and clinical study. Signal Transduct Target Ther (2020) 5(1):1–23. doi: 10.1038/s41392-020-00312-6 32958760 PMC7506548

[72] Skov J Persson F Frøkiær J Christiansen JS. Tissue renin-angiotensin systems: A unifying hypothesis of metabolic disease. Front Endocrinol (Lausanne) (2014) 5:1–7. doi: 10.3389/fendo.2014.00023 24592256 PMC3938116

[73] Froogh G Kandhi S Duvvi R Le Y Weng Z Alruwaili N. The contribution of chymase-dependent formation of ANG II to cardiac dysfunction in metabolic syndrome of young rats: Roles of fructose and EETs. Am J Physiol - Hear Circ Physiol (2020) 318(4):985–93. doi: 10.1152/ajpheart.00633.2019 PMC7191492 32167781

[74] Singh VP Le B Khode R Baker KM Kumar R. Intracellular angiotensin II production in diabetic rats is correlated with cardiomyocyte apoptosis, oxidative stress, and cardiac fibrosis. Diabetes (2008) 57(12):3297–306. doi: 10.2337/db08-0805 PMC2584136 18829990

[75] Ferrario SA Ahmad S. Chymase inhibitors for the treatment of cardiac diseases: A patent review (2010-2018. Expert Opin Ther Pat (2018) 28(11):755–64. doi: 10.1080/13543776.2018.1531848 PMC6240413 30278800

[76] Maeda Y Inoguchi T Takei R Sawada F Sasaki S Fujii M. Inhibition of chymase protects against diabetes-induced oxidative stress and renal dysfunction in hamsters. Am J Physiol - Ren Physiol (2010) 299(6):1328–38. doi: 10.1152/ajprenal.00337.2010 20881036

[77] Arun KHS Kaul CL Ramarao P. High glucose concentration augments angiotensin-II mediated contraction *via* AT1 receptors in rat thoracic aorta. Pharmacol Res (2004) 50(6):561–8. doi: 10.1016/j.phrs.2004.06.001 15501693

[78] Wang GY Bi YG Liu XD Zhao Y Han JF Wei M. Autophagy was involved in the protective effect of metformin on hyperglycemia-induced cardiomyocyte apoptosis and connexin43 downregulation in H9c2 cells. Int J Med Sci (2017) 14(7):698–704. doi: 10.7150/ijms.19800 28824303 PMC5562122

[79] Yamagishi SI Matsui T. Advanced glycation end products, oxidative stress and diabetic nephropathy. Oxid Med Cell Longev (2010) 3(2):101–8. doi: 10.4161/oxim.3.2.11148 PMC2952094 20716934

[80] Koka V Wang W Xiao RH Shokei KM Truong LD Lan HY. Advanced glycation end products activate a chymase-dependent angiotensin II-generating pathway in diabetic complications. Circulation (2006) 113(10):1353–60. doi: 10.1161/CIRCULATIONAHA.105.575589 PMC1401500 16520412

[81] Sasa V Kathy G. Angiotensin II, from vasoconstrictor to growth factor: A paradigm shift. Circ Res (2014) 114(5):754–7. doi: 10.1161/circresaha.114.303045 PMC3985550 24577962

[82] Ichimiya T Yamakawa T Hirano T Yokoyama Y Hayashi Y Hirayama D. Autophagy and autophagy-related diseases: A review. Int J Mol Sci (2020) 21(23):8974. doi: 10.3390/ijms21238974 PMC7729615 33255983

[83] Parzych KR Klionsky DJ. An overview of autophagy: Morphology, mechanism, and regulation. Antioxid Redox Signal (2014) 20(3):460–73. doi: 10.1089/ars.2013.5371 PMC3894687 23725295

[84] Wu D Zhang K Hu P. The role of autophagy in acute myocardial infarction. Front Pharmacol (2019) 10:551. doi: 10.3389/fphar.2019.00551 31214022 PMC6554699

[85] Yim WWY Mizushima N. Lysosome biology in autophagy. Cell Discov [Internet] (2020) 6(1):1–12. doi: 10.1038/s41421-020-0141-7 PMC7010707 32047650

[86] Nishimura T Tooze SA. Emerging roles of ATG proteins and membrane lipids in autophagosome formation. Cell Discov (2020) 6(1):1–18. doi: 10.1038/s41421-020-0161-3 32509328 PMC7248066

[87] Gallagher LE Williamson LE Chan EYW. Advances in autophagy regulatory mechanisms. Cells (2016) 5(2):24. doi: 10.3390/cells5020024 PMC4931673 27187479

[88] Zachari M Ganley IG. The mammalian ULK1 complex and autophagy initiation. Essays Biochem (2017) 61(6):585–96. doi: 10.1042/ebc20170021 PMC5869855 29233870

[89] Al-Bari MAA Xu P. Molecular regulation of autophagy machinery by mTOR-dependent and -independent pathways. Ann NY Acad Sci (2020) 1467(1):3–20. doi: 10.1111/nyas.14305 31985829

[90] Tamargo-Gómez I Mariño G. AMPK: Regulation of metabolic dynamics in the context of autophagy. Int J Mol Sci (2018) 19(12):3812. doi: 10.3390/ijms19123812 PMC6321489 30501132

[91] Dewanjee S Vallamkondu J Kalra RS John A Reddy PH Kandimalla R. Autophagy in the diabetic heart: A potential pharmacotherapeutic target in diabetic cardiomyopathy. Ageing Res Rev (2021) 68:101338. doi: 10.1016/j.arr.2021.101338 33838320

[92] Kanamori H Takemura G Goto K Tsujimoto A Mikami A Ogino A. Autophagic adaptations in diabetic cardiomyopathy differ between type 1 and type 2 diabetes. Autophagy (2015) 11(7):1146–60. doi: 10.1080/15548627.2015.1051295 PMC4590644 26042865

[93] Shao D Tian R. Glucose transporters in cardiac metabolism and hypertrophy. Compr Physiol (2016) 6(1):331–51. doi: 10.1002/cphy.c150016 PMC4760112 26756635

[94] Munasinghe P Katare R. Maladaptive autophagy in diabetic heart disease. Int J Clin Exp Physiol (2016) 3(4):155–65. doi: 10.4103/2348-8832.196893

[95] Kobayashi S Liang Q. Autophagy and mitophagy in diabetic cardiomyopathy. Biochim Biophys Acta - Mol Basis Dis (2015) 1852(2):252–61. doi: 10.1016/j.bbadis.2014.05.020 24882754

[96] Li X Ke X Li Z Li B. Vaspin prevents myocardial injury in rats model of diabetic cardiomyopathy by enhancing autophagy and inhibiting inflammation. Biochem Biophys Res Commun (2019) 514(1):1–8. doi: 10.1016/j.bbrc.2019.04.110 31014675

[97] Xu X Kobayashi S Chen K Timm D Volden P Huang Y. Diminished autophagy limits cardiac injury in mouse models of type 1 diabetes. J Biol Chem (2013) 288(25):18077–92. doi: 10.1074/jbc.M113.474650 PMC3689952 23658055

[98] Bhattacharya D Mukhopadhyay M Bhattacharyya M Karmakar P. Is autophagy associated with diabetes mellitus and its complications? A review. EXCLI J (2018) 17:709–20. doi: 10.17179/excli2018-1353 PMC6123605 30190661

[99] Kanamori H Naruse G Yoshida A Minatoguchi S Watanabe T Kawaguchi T. Morphological characteristics in diabetic cardiomyopathy associated with autophagy. J Cardiol (2021) 77(1):30–40. doi: 10.1016/j.jjcc.2020.05.009 32907780

[100] Kobayashi S Xu X Chen K Liang Q. Suppression of autophagy is protective in high glucose-induced cardiomyocyte injury. Autophagy (2012) 8(4):577–92. doi: 10.4161/auto.18980 PMC3405845 22498478

[101] Ouyang C You J Xie Z. The interplay between autophagy and apoptosis in the diabetic heart. J Mol Cell Cardiol (2014) 71:71–80. doi: 10.1016/j.yjmcc.2013.10.014 24513079

[102] Venkatesh I Makky K. Teaching epigenetic regulation of gene expression is critical in 21st-century science education: Key concepts & teaching strategies. Am Biol Teach (2020) 82(6):372–80. doi: 10.1525/abt.2020.82.6.372

[103] Aristizabal MJ Anreiter I Halldorsdottir T Odgers CL McDade TW Goldenberg A. Biological embedding of experience: A primer on epigenetics. Proc Natl Acad Sci USA (2020) 117(38):23261–9. doi: 10.1073/pnas.1820838116 PMC7519272 31624126

[104] Zheng J Cheng J Zhang Q Xiao X. Novel insights into DNA methylation and its critical implications in diabetic vascular complications. Biosci Rep (2017) 37(2):1–8. doi: 10.1042/BSR20160611 PMC5350598 28183874

[105] Pepin ME Wende AR. Epigenetics in the development of diabetic cardiomyopathy. Epigenomics (2019) 11(5):469–72. doi: 10.2217/epi-2019-0027 PMC12834566 30895816

[106] Liu Z Zhang Y Qiu C Zhu H Pan S Jia H. Diabetes mellitus exacerbates post-myocardial infarction heart failure by reducing sarcolipin promoter methylation. ESC Hear Fail (2020) 7(4):1935–48. doi: 10.1002/ehf2.12789 PMC7373908 32525286

[107] Tsai CT Wu CK Lee JK Chang SN Kuo YM Wang YC. TNF-α down-regulates sarcoplasmic reticulum Ca2+ ATPase expression and leads to left ventricular diastolic dysfunction through binding of NF-κB to promoter response element. Cardiovasc Res (2015) 105(3):318–29. doi: 10.1093/cvr/cvv008 25712896

[108] Madsen A Höppner G Krause J Hirt MN Laufer SD Schweizer M. An important role for DNMT3a-mediated DNA methylation in cardiomyocyte metabolism and contractility. Circulation (2020) 142(16):1562–78. doi: 10.1161/CIRCULATIONAHA.119.044444 PMC7566310 32885664

[109] Yerra VG Advani A. Histones and heart failure in diabetes. Cell Mol Life Sci [Internet] (2018) 75(17):3193–213. doi: 10.1007/s00018-018-2857-1 PMC6063320 29934664

[110] Qi YK Ai HS Li YM Yan B. Total chemical synthesis of modified histones. Front Chem (2018) 6(19):1–11. doi: 10.3389/fchem.2018.00019 29473034 PMC5810247

[111] Demetriadou C Koufaris C Kirmizis A. Histone n-alpha terminal modifications: Genome regulation at the tip of the tail. Epigenet Chromatin (2020) 13(1):1–13. doi: 10.1186/s13072-020-00352-w PMC7367250 32680559

[112] Hussain S Khan AW Akhmedov A Suades R Costantino S Paneni F. Hyperglycemia induces myocardial dysfunction *via* epigenetic regulation of JunD. Circ Res (2020) 127(10):1261–73. doi: 10.1161/CIRCRESAHA.120.317132 32815777

[113] Villeneuve LM Natarajan R. The role of epigenetics in the pathology of diabetic complications. Am J Physiol - Ren Physiol (2010) 299(1):14–25. doi: 10.1152/ajprenal.00200.2010 PMC2904177 20462972

[114] Ke X Lin Z Ye Z Leng M Chen B Jiang C. Histone deacetylases in the pathogenesis of diabetic cardiomyopathy. Front Endocrinol (Lausanne) (2021) 12:889. doi: 10.3389/fendo.2021.679655 PMC8339406 34367065

[115] Deng J Liao Y Liu J Liu W Yan D. Research progress on epigenetics of diabetic cardiomyopathy in type 2 diabetes. Front Cell Dev Biol (2021) 9:1–10. doi: 10.3389/fcell.2021.777258 PMC8740193 35004678

[116] O’Brien J Hayder H Zayed Y Peng C. Overview of microRNA biogenesis, mechanisms of actions, and circulation. Front Endocrinol (Lausanne) (2018) 9:402. doi: 10.3389/fendo.2018.00402 30123182 PMC6085463

[117] Rawal S Ram TP Coffey S Williams MJA Saxena P Bunton RW. Differential expression pattern of cardiovascular microRNAs in the human type-2 diabetic heart with normal ejection fraction. Int J Cardiol (2016) 202:40–3. doi: 10.1016/j.ijcard.2015.08.161 26386917

[118] Mann VD Mann DL. The emerging role of MicroRNAs in cardiac remodeling and heart failure. Circ Res (2008) 103(10):1072–83. doi: 10.1161/circresaha.108.183087 PMC3982911 18988904

[119] Nair RG Nair S. Role of MicroRNA in diabetic cardiomyopathy: from mechanism to intervention. Biochim Biophys Acta (2017) 1863(8):2070–7. doi: 10.1016/j.bbadis.2017.03.013 PMC5475364 28344129

[120] Liu X Liu S. Role of microRNAs in the pathogenesis of diabetic cardiomyopathy. BioMed Rep (2017) 6(2):140–5. doi: 10.3892/br.2017.841 PMC5351040 28357065

[121] Shantikumar S Caporali A Emanueli C. Role of microRNAs in diabetes and its cardiovascular complications. Cardiovasc Res (2012) 93(4):583–93. doi: 10.1093/cvr/cvr300 PMC3291087 22065734

[122] Ahmed U Ashfaq UA Qasim M Ahmad I Ahmad HU Tariq M. Dysregulation of circulating miRNAs promotes the pathogenesis of diabetes-induced cardiomyopathy. PloS One (2021) 16(4):1–14. doi: 10.1371/journal.pone.0250773 PMC8081166 33909697

[123] Li H Fan J Zhao Y Zhang X Dai B Zhan J. Nuclear miR-320 mediates diabetes-induced cardiac dysfunction by activating transcription of fatty acid metabolic genes to cause lipotoxicity in the heart. Circ Res (2019) 125(12):1106–20. doi: 10.1161/CIRCRESAHA.119.314898 PMC6903355 31638474

[124] Moorthy S Koshy T Srinivasan V Silambanan S. Circulating biomarkers and MicroRNAs in the diagnosis, prognosis and treatment of diabetic cardiomyopathy-a review. Cardiol Cardiovasc Med (2020) 04(04):481–97. doi: 10.26502/fccm.92920145

[125] Dai B Li H Fan J Zhao Y Yin Z Nie X. MiR-21 protected against diabetic cardiomyopathy induced diastolic dysfunction by targeting gelsolin. Cardiovasc Diabetol (2018) 17(1):1–17. doi: 10.1186/s12933-018-0767-z 30180843 PMC6122727

[126] Asrih M Steffens S. Emerging role of epigenetics and miRNA in diabetic cardiomyopathy. Cardiovasc Pathol (2013) 22(2):117–25. doi: 10.1016/j.carpath.2012.07.004 22951386

